# Supplementary material for: Proteomic analysis of archival breast cancer clinical specimens identifies biological subtypes with distinct survival outcomes
Source: Nat Commun. 2022 Feb 16;13:896. doi: 10.1038/s41467-022-28524-0 (PMC8850446; doi:10.1038/s41467-022-28524-0)
Supplement: Supplementary file 1 — Supplementary Information [file 41467_2022_28524_MOESM1_ESM.pdf]

Supplementary Information

Proteomic analysis of archival breast cancer clinical specimens identifies biological subtypes with distinct survival outcomes

Supplementary Information includes:

1. Supplementary Figures (pages 2-27)
2. Supplementary Tables (pages 28-30)
3. Supplementary References (page 31)

Supplementary Figures:

Supplementary Figure 1

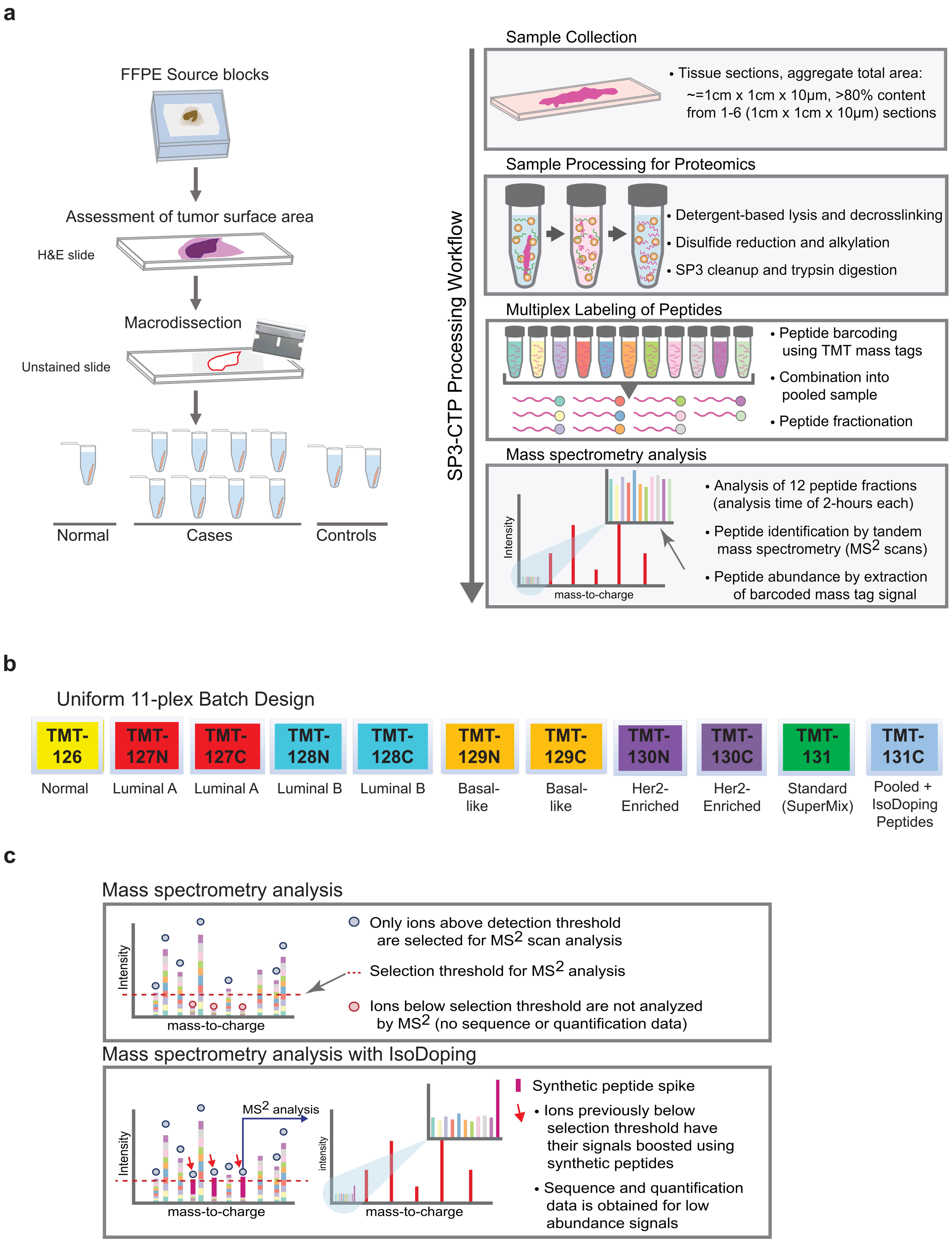

Supplementary Figure 1. Patient samples and study workflow.

(a) FFPE samples were macrodissected and analyzed using the highly sensitive SP3-CTP multiplex mass spectrometry proteomics protocol. Digested peptides were labeled with a stable isotope labeled TMT and run in 11-plex TMT sample sets. (b) The uniform batch design for the 38 x 11-plex TMT sets. Subtypes were split evenly among plexes and each plex included one normal, a SuperMix standard, and a pooled internal standard into which the IsoDoping peptides were added. (c) Cartoon showing mass spectrometry analysis using TMT-based MS2 for global proteome profiling (top panel) and for when the isobaric peptide doping (IsoDoping) strategy is used (bottom panel). Abbreviations: TNBC, triple-negative breast cancer; IHC, immunohistochemistry; FFPE, Formalin-fixed paraffin-embedded; H&E, hematoxylin and eosin; SP3, Single-Pot, Solid-Phase-enhanced, Sample Preparation; CTP, clinical tissue proteomics; TMT, tandem mass tag. Source data are provided as a Source Data file.

Supplementary Figure 2

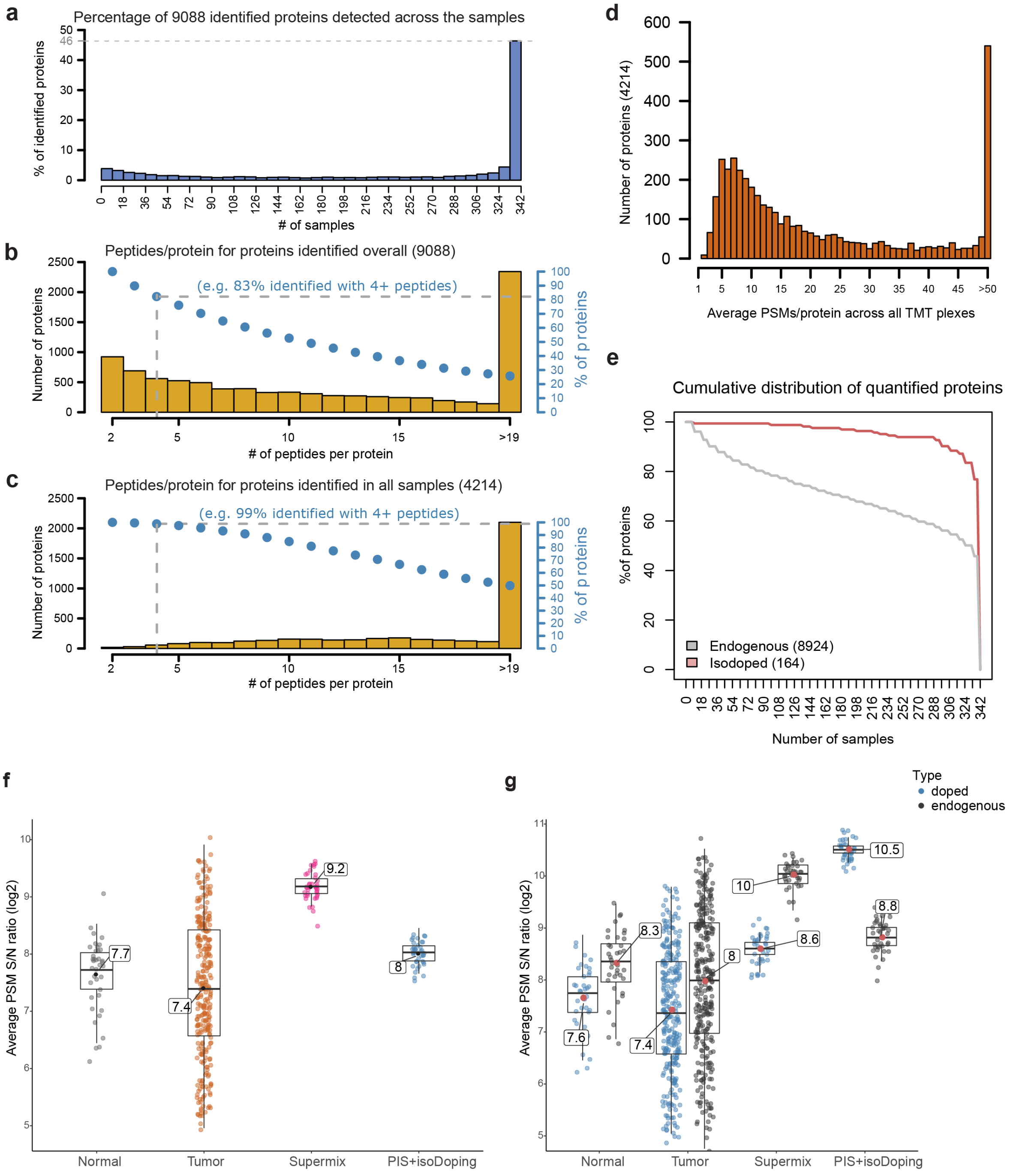

Supplementary Figure 2. Mass spectrometry analysis and performance of peptide quantification according to sample type.

(a) Percentage of the total number of proteins detected in different number of samples.

(b and c) Numbers and percentages of the total number of proteins detected in different number of samples according to number of peptides per protein. Yellow bars in the histogram show the number of proteins identified by different number of peptides per protein. Blue dots show the percentage of total proteins identified per minimal number of peptides per protein. The (b) and (c) plots are for the 9088 and 4214 proteins identified overall and across all samples, respectively. (d) Average number of quantified PSMs per protein, across the full cohort (corresponding to the 4214 proteins quantified across all samples). (e) Cumulative distribution of the percentage of isoDoped and endogenous proteins across the tumor cohort. (f) Boxplots showing the unnormalized average PSM signal to noise ratio by sample type with an average difference of 3.7x between SuperMix and tumor samples, and with all SuperMix samples showing an average S/N comparable to the tumor samples with higher signal. Boxplots show the median (center bar), and the third and first quartiles (upper and lower edges, respectively) of protein expression. Boxplot whiskers range extends to the most extreme data point which is no more than 1.5 times the interquartile range from the box. Sample sizes for each group presented are as follows: normal (n=38), tumor (n=304), SuperMix (n=38) and PIS (n=38). (g) Boxplots showing a comparison between the unnormalized average PSM S/N ratio for isoDoped peptides vs. endogenous peptides, for the isoDoped proteins, by sample type. The plot displays that there is only a 3.2x difference between the average abundance of isoDoped peptides and endogenous peptides for the isodoped proteins in the PIS+isoDoping channel. Comparing the average S/N of the isoDoping peptides in the tumor samples and the spiked-in channel (PIS+isoDoped), detected

an 8.6x difference, which is below the suggested limit of 20x (Cheung TK et al. *Nature Methods* 2021[1]). Boxplots show the median (center bar), and the third and first quartiles (upper and lower edges, respectively) of protein expression. Boxplot whiskers range extends to the most extreme data point which is no more than 1.5 times the interquartile range from the box. Sample sizes for each group presented are as follows: normal (n=38), tumor (n=304), SuperMix (n=38) and PIS (n=38). Average S/N were calculated over PSMs matching isoDoped peptides (n=665) or endogenous peptides (n=1753) matching isoDoped proteins. Source data are provided as a Source Data file.

Supplementary Figure 3

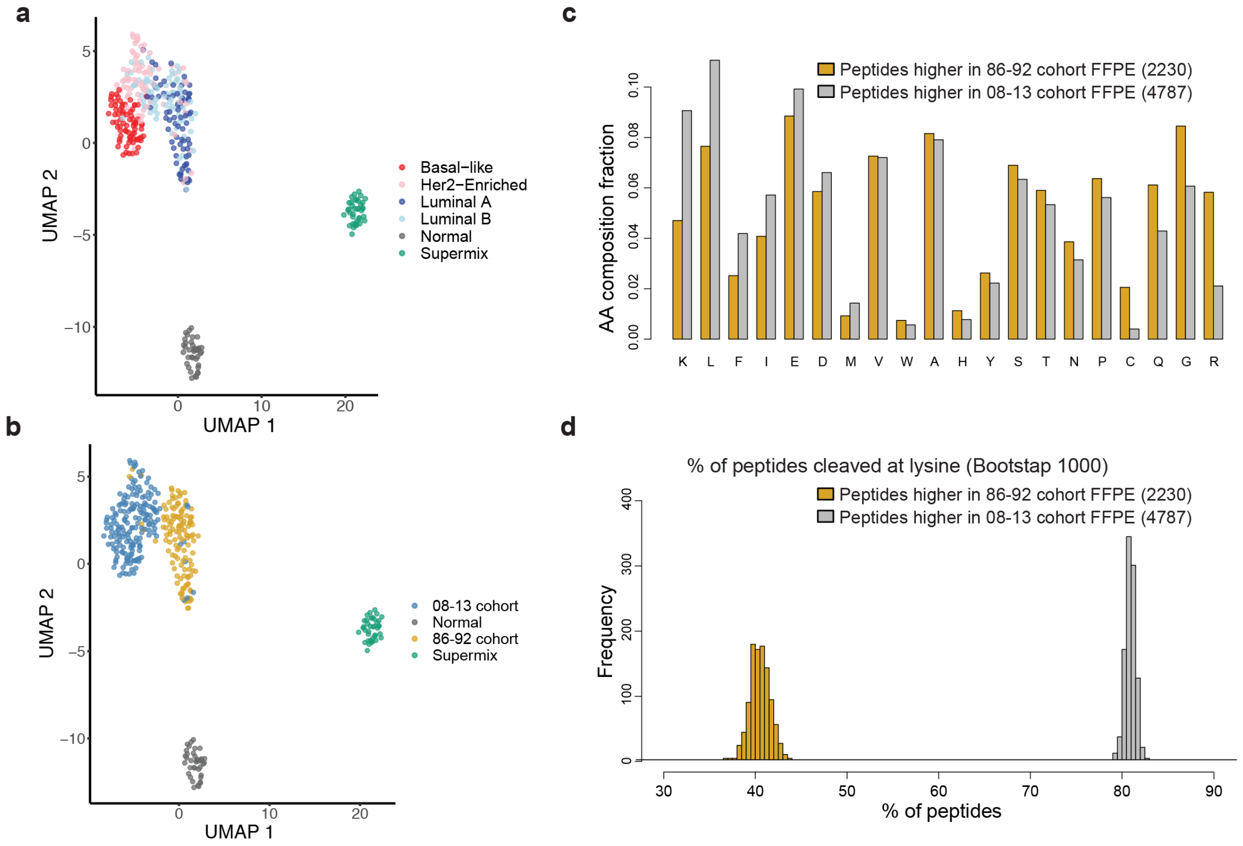

Supplementary Figure 3. Mass spectrometry analysis of study cohorts according to set characteristics.

(a and b) Uniform Manifold Approximation and Projection showing the separation of total samples according to (a) set characteristics (b) time of initial sample collection. (c) Distribution of amino acid composition fraction of peptides showing differential abundance (absolute fold change >1.2, p-value <0.05) between the 08-13 vs 86-92 Luminal B samples. Results are derived from peptide-level expression-change averaging (PECA) analysis performed at the peptide level, using modified t-test and results were adjusted for multiple comparisons using the Benjamini-Hochberg method. (d) Bootstrapped distribution of percentage of peptides cleaved at lysine residue showing differential abundance (absolute fold change >1.2, p-value <0.05) between the 08-13 vs 86-92 Luminal B samples. Results are derived from peptide-level expression-change

averaging (PECA) analysis performed at the peptide level, using modified t-test and results were adjusted form multiple comparisons using the Benjamini-Hochberg method. Source data are provided as a Source Data file.

Supplementary Figure 4

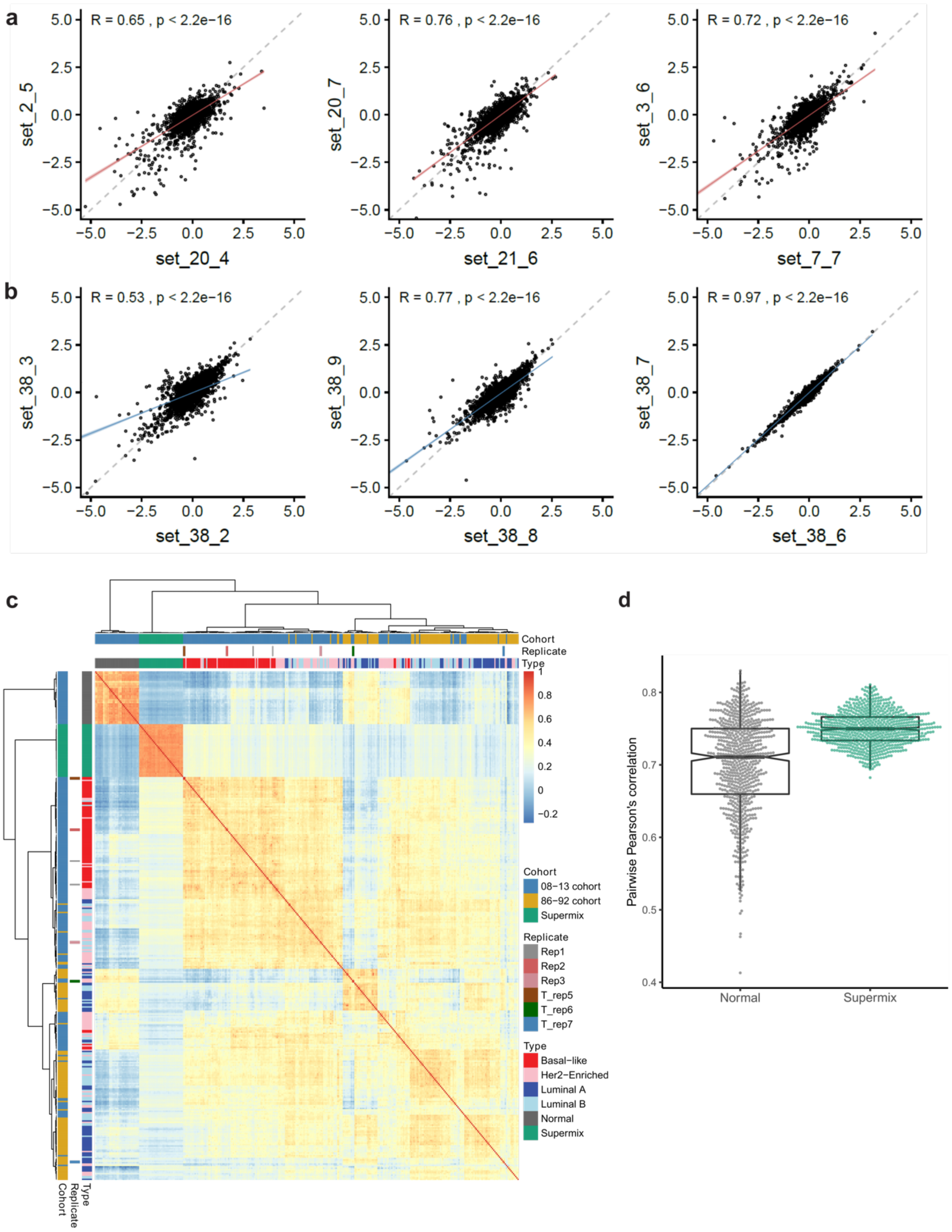

Supplementary Figure 4. Reproducibility and robust TMT quantification across the study sample sets. (a) Reproducibility between 3 biological replicates as indicated by a 2-sided Pearson's product-moment correlation test of protein expression values. (b) Reproducibility between 3 technical replicates as indicated by a 2-sided Pearson's product-moment correlation test of protein expression values. (c) An overview clustering for all the samples including breast tumors, normals, and SuperMix. All the 38 SuperMix samples are clustered together and clearly separated from the rest of other breast samples. The correlation between the SuperMix samples is shown to be the highest when compared to breast tumor and normal samples. (d) Boxplots showing the pairwise correlation between the 38 SuperMix replicates ranging between 0.68-0.81 (median 0.75) when compared to the correlation observed for the 38 normals across the plexes ranging between 0.53- 0.85 (median 0.71). Boxplots show the median (center bar), and the third and first quartiles (upper and lower edges, respectively) of protein expression. Boxplot whiskers range extends to the most extreme data point which is no more than 1.5 times the interquartile range from the box. Source data are provided as a Source Data file.

Supplementary Figure 5

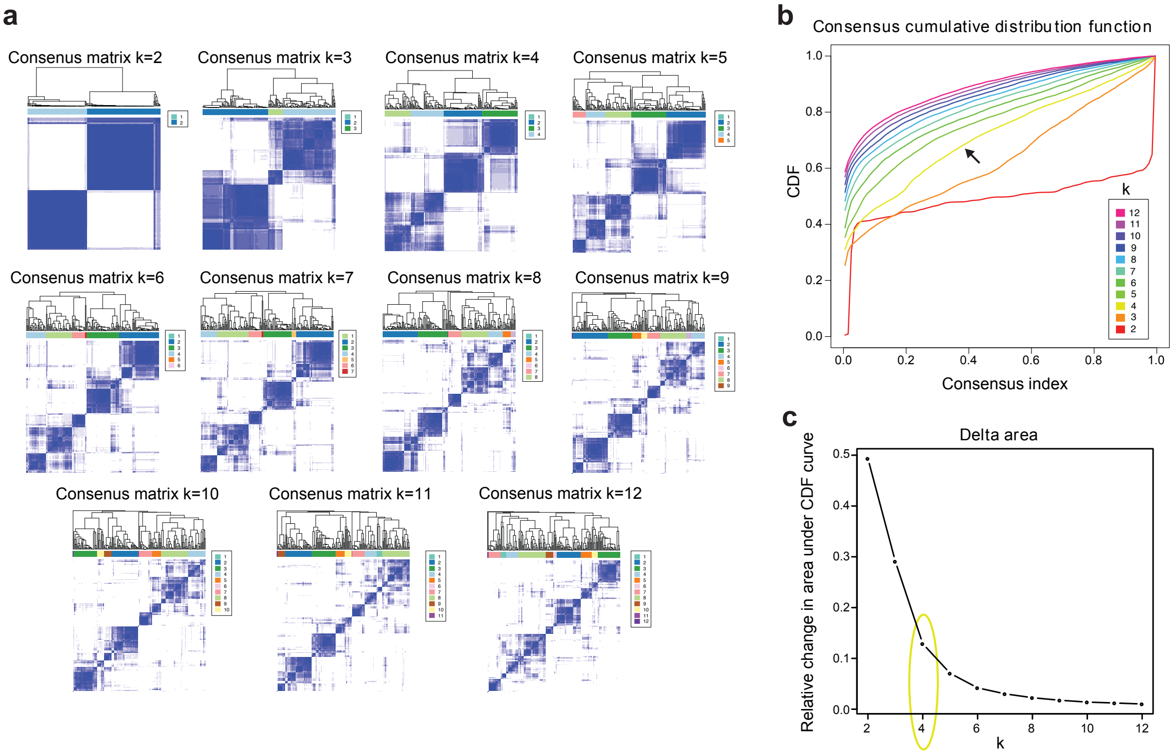

Supplementary Figure 5. Robust segregation of samples in the 08-13 cohort.

(a) Consensus matrices exploring the range of 2 to 12 *K*-means clusters for tumors in the 08-13 cohort using consensus clustering on the 1054 most variant proteins. (b) consensus CDF area. (c) Delta area showing the relative change in area under the CDF curve. Four robustly segregated groups displayed a clear separation of the clusters based on visual inspection and largest change in area under the CDF curve in delta plot when exploring the range of 2 to 12 *K*-means clusters. Abbreviations: CDF, cumulative distribution function. Source data are provided as a Source Data file.

Supplementary Figure 6

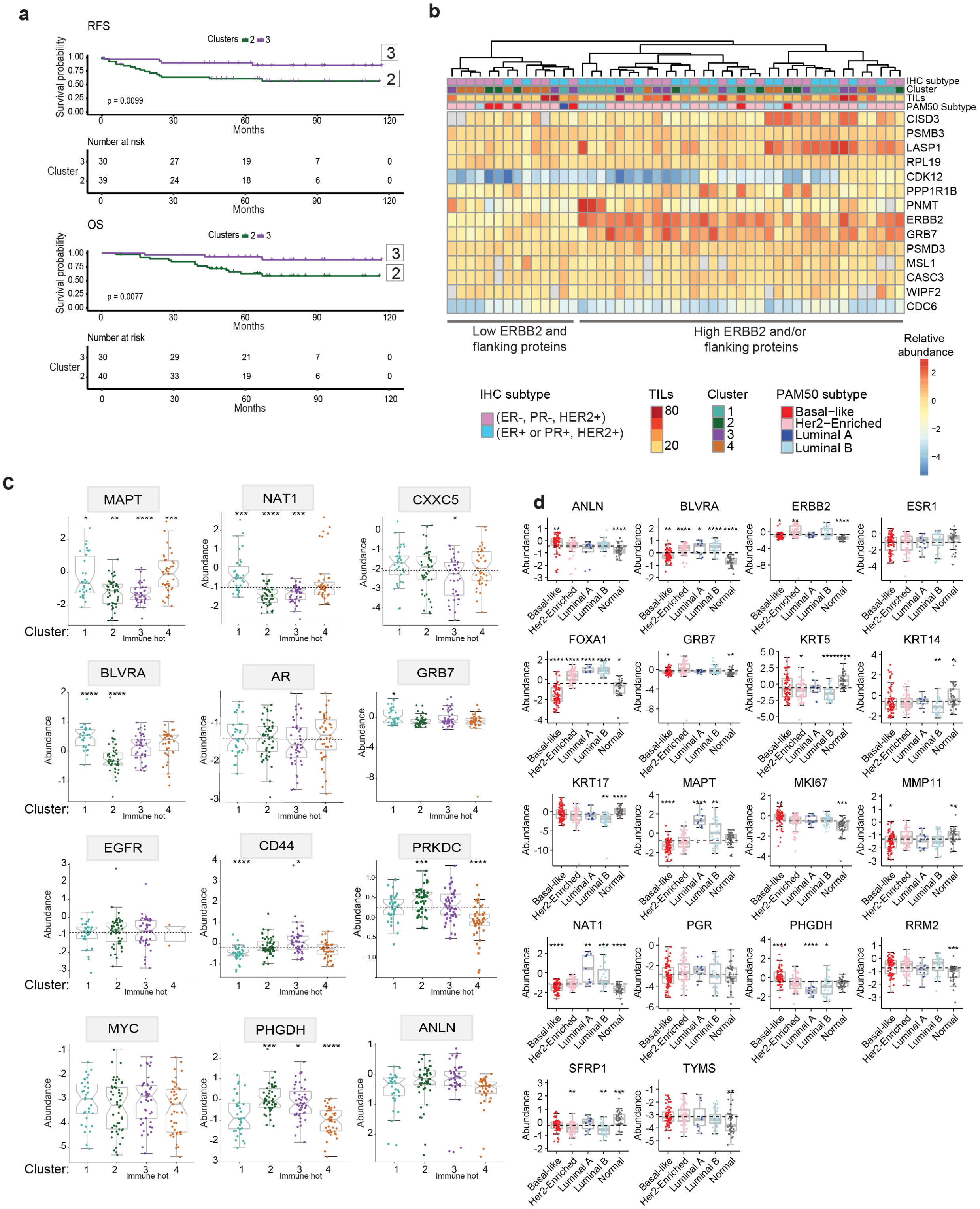

Supplementary Figure 6. Characteristics of proteome breast cancer clusters in the 08-13 cohort.

(a) Kaplan Meier plots show distinct clinical outcomes of RFS and OS for basal-like PAM50 subtype Cluster-3 cases only vs. basal-like cases only in Cluster-2 of the 08-13 cohort. (b) Heatmap showing the proteomic expression of ERBB2 and proteins for flanking genes in the *ERBB2* amplicon among 49 cases classified as clinically Her2+ breast cancer. Proteomic profiling identified a subset of cases with an overall low abundance of ERBB2 and its flanking proteins (Low ERBB2 and flanking proteins). A subset of cases with overexpression for ERBB2 or other flanking proteins for the *ERBB2* amplicon was also identified (High ERBB2 and/or flanking proteins). (c) Expression of key subtype specific breast cancer proteins across the different proteome clusters in 08-13 cohort. Boxplots show the median (center bar), and the third and first quartiles (upper and lower edges, respectively). Boxplot whiskers range extends to the most extreme data point which is no more than 1.5 times the interquartile range from the box. Asterisks show the pairwise significance of the mean in each group against "all" as a reference: *(p<0.05), **(p<0.01), ***(p<0.001), ****(p<0.0001). (d) Expression of selected key subtype specific breast cancer proteins of the PAM50 signature across the different PAM50 subtypes and normal cases in the 08-13 cohort. Boxplots show the median (center bar), and the third and first quartiles (upper and lower edges, respectively). Boxplot whiskers range extends to the most extreme data point which is no more than 1.5 times the interquartile range from the box. Asterisks show the pairwise significance of the mean in each group against "all" as a reference: *(p<0.05), **(p<0.01), ***(p<0.001), ****(p<0.0001). Abbreviations: RFS, recurrence free survival; OS, overall survival. Related to Figure 3. Source data are provided as a Source Data file.

Supplementary Figure 7

a

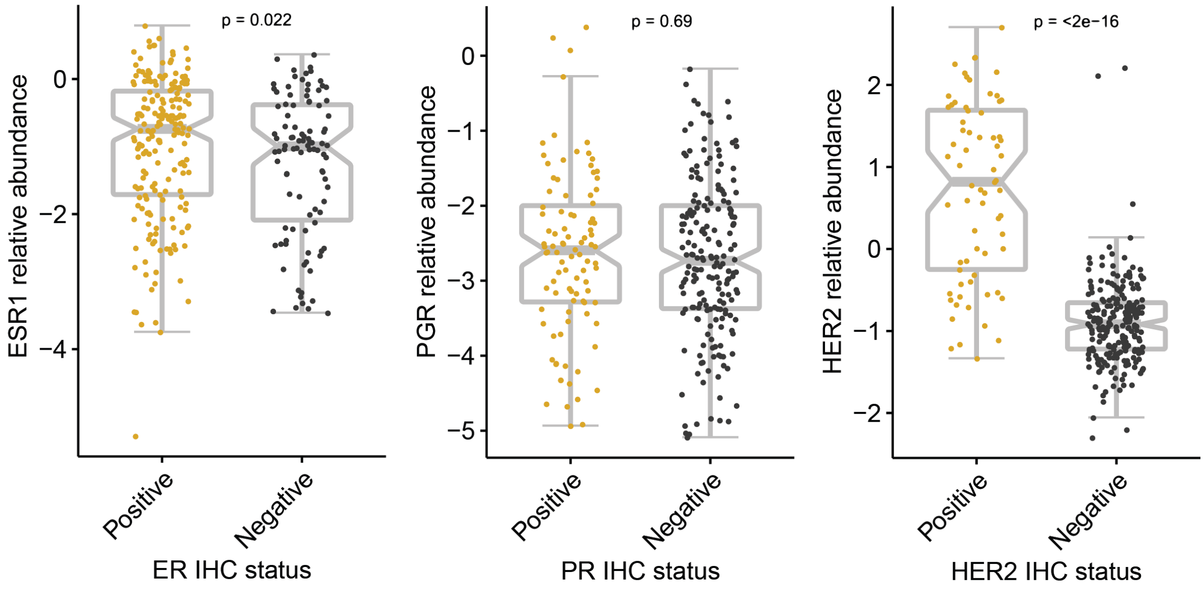

b

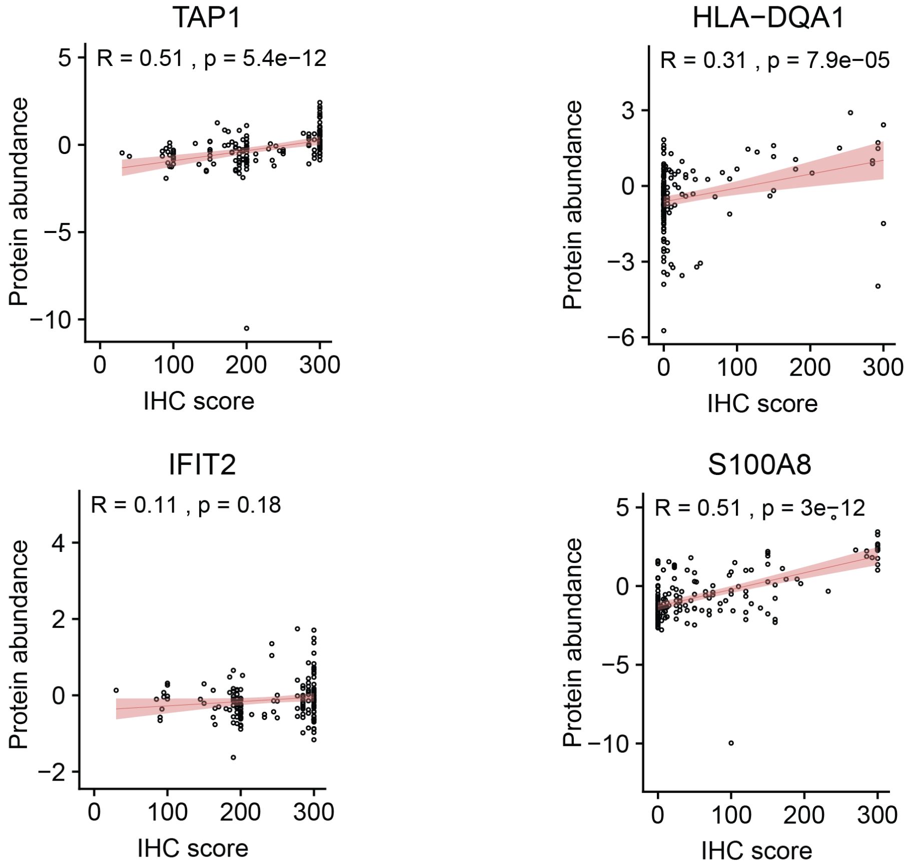

Supplementary Fig. 7. Correlation between proteomic abundance scores vs. immunohistochemistry for selected proteins.

(a) Relative abundance of ESR1, PGR and HER2 by Mass spectrometry according their IHC categories. Boxplots show the median (center bar), and the third and first quartiles (upper and lower edges, respectively). Boxplot whiskers range extends to the most extreme data point which is no more than 1.5 times the interquartile range from the box. Results are derived from a Wilcoxon test with a 2-sided p-value. (b) Correlation of protein expression values for protein candidates by mass spectrometry vs. IHC values. The error bands represented with a red area correspond to 95% confidence interval. Scoring of the S100A8, TAP1, IFIT2 and HLA-DQA1 IHC biomarkers were reported using the H scoring system (intensity x positivity) for the cytoplasmic staining observed in the invasive breast tumor cells. Spearman correlations are shown on each panel. Abbreviations: IHC, immunohistochemistry. Source data are provided as a Source Data file.

Supplementary Figure 8

a

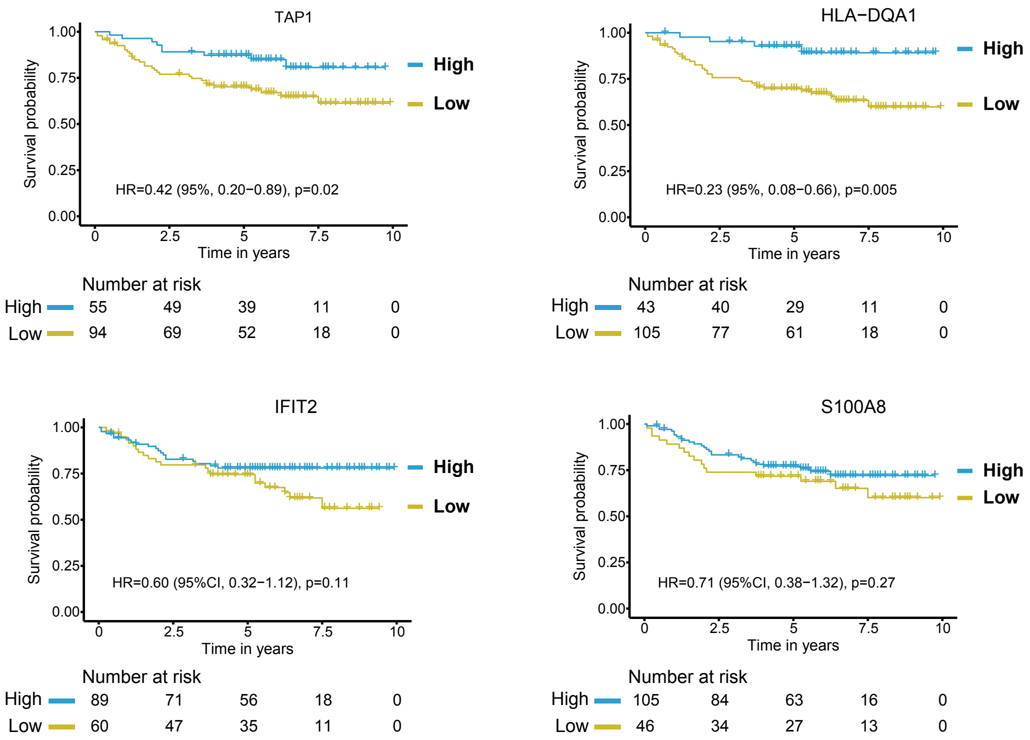

b

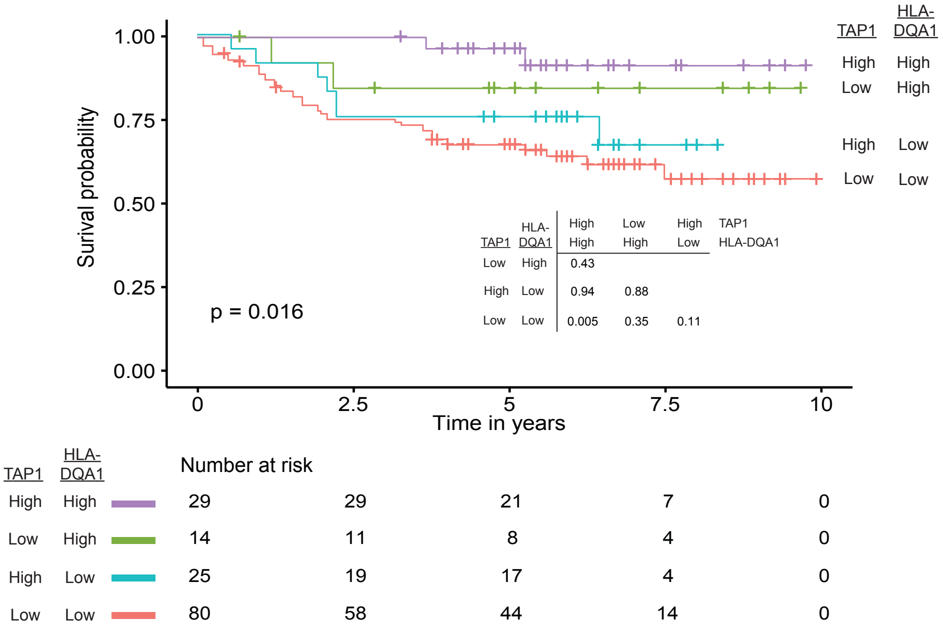

Supplementary Figure 8. Prognostic value of selected protein candidates enriched in Cluster-3 (immune hot) of the 08-13 cohort.

(a) Kaplan-Meier survival curves showing RFS stratified according to individual biomarker IHC expression categories. H-scores were dichotomized using cut-points optimized for best Cox model fit to define high vs. low categories. (b) RFS for cases further stratified according to the combinatorial IHC expression of TAP1 and HLA-DQA1 categories. Abbreviations: RFS, recurrence free survival; IHC, immunohistochemistry. Source data are provided as a Source Data file.

Supplementary Figure 9

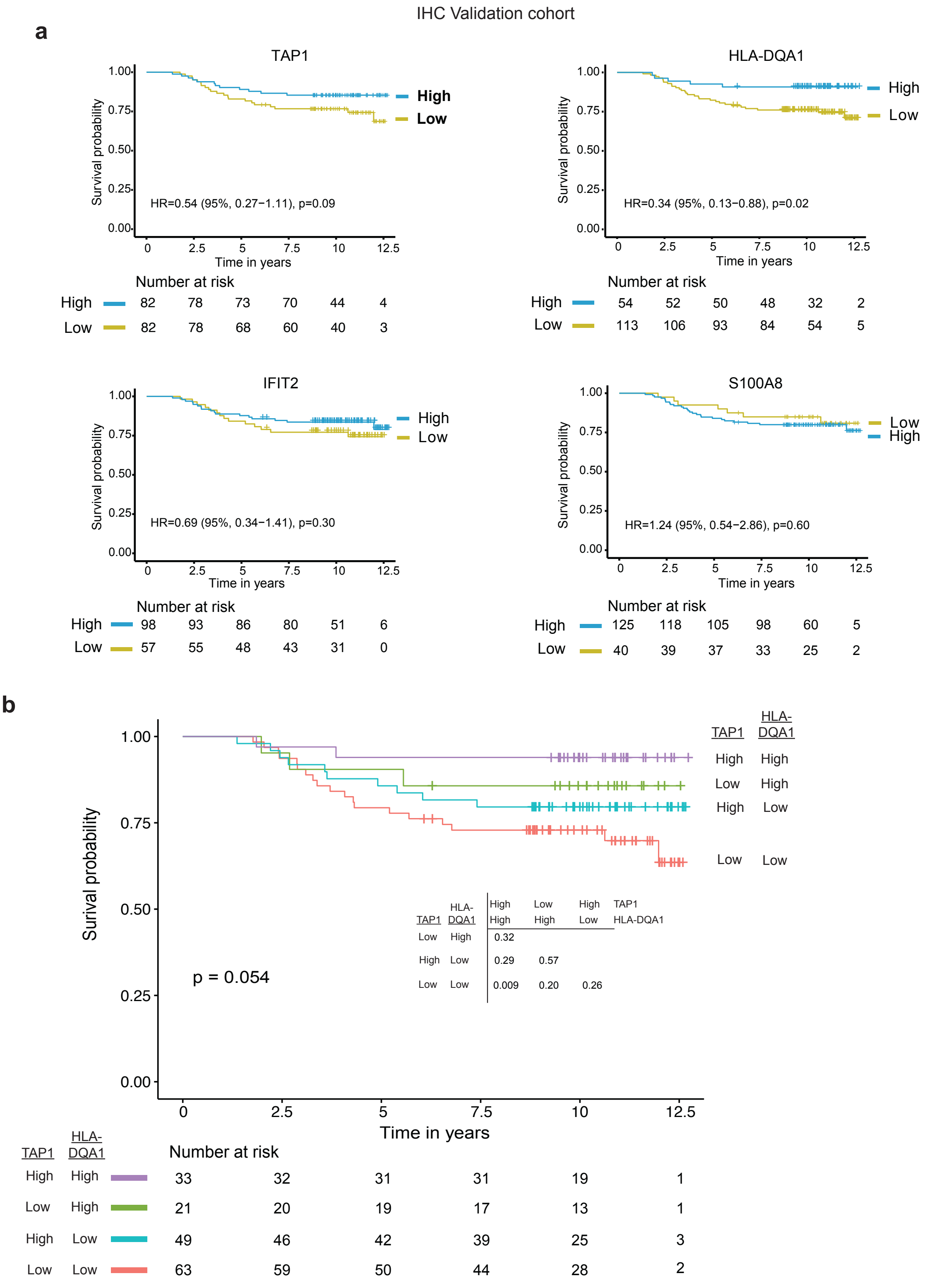

Supplementary Figure 9. Validation of the prognostic value of selected IHC protein candidates enriched in Cluster-3 (immune hot) of the 08-13 cohort using a similar independent set of 176 breast cancer cases. (a) Kaplan-Meier survival curves stratified according to individual biomarker IHC expression categories using the prespecified dichotomized cut-points optimized from the best Cox model fit on the 08-13 cohort to define high vs. low categories. (b) Kaplan-Meier survival curves for cases further stratified according to the combinatorial IHC expression of TAP1 and HLA-DQA1 categories. Abbreviations: RFS, recurrence free survival; IHC, immunohistochemistry. Source data are provided as a Source Data file.

Supplementary Figure 10

a) Validation using the Krug et al. 2020 CPTAC breast tumor cohort

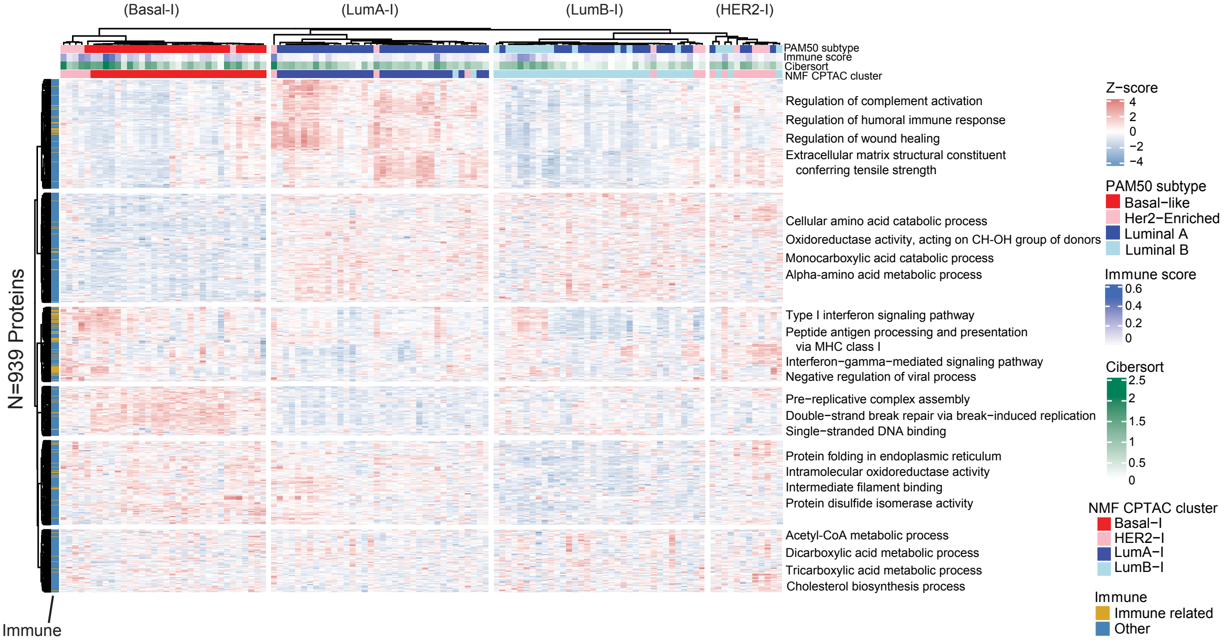

b) Validation using the Johansson et al 2019 OSLO2 breast cancer landscape cohort

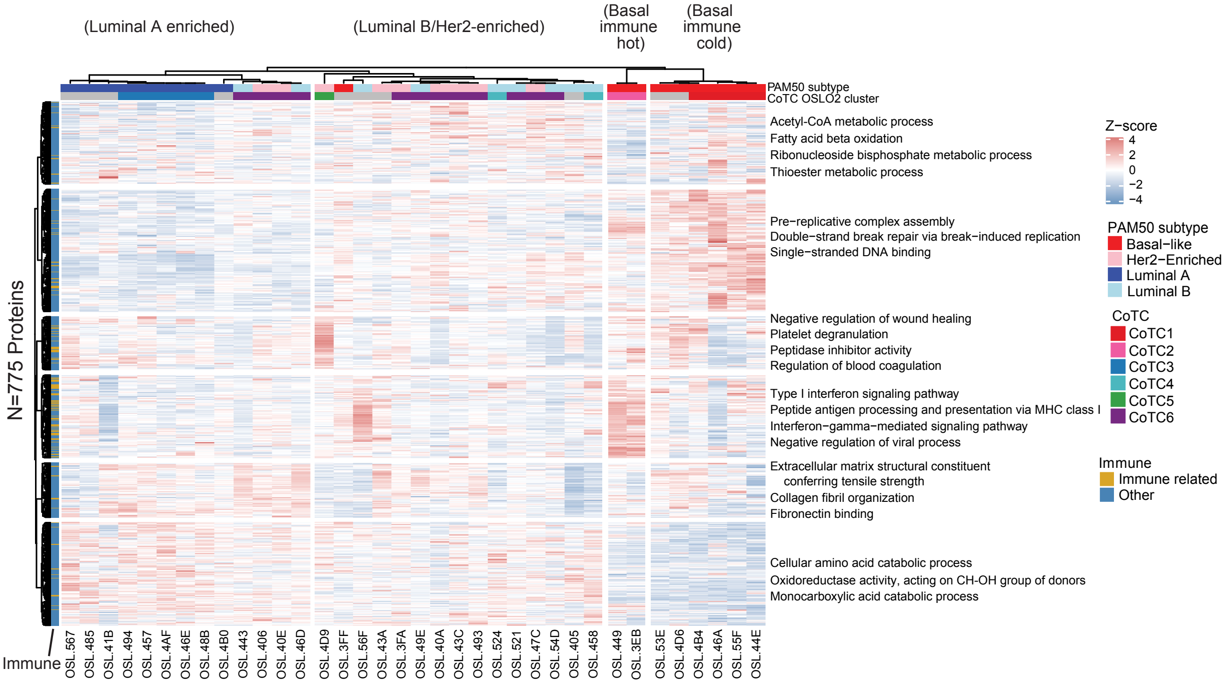

Supplementary Figure 10. Comparison with previous proteomics breast cancer datasets. (a) Validation using the Krug et al. 2020 CPTAC breast tumor cohort: In order to compare our results with available published datasets, we performed consensus clustering with the same parameters used in our cohort on the CPTAC *Cell* 2020 cohort, using the 939 proteins from the CPTAC data that overlap with the 1054 mostly highly-variant proteins of our 08-13 cohort. This analysis identified four main proteome clusters that highly resembled the original CPTAC NMF clusters of LumA-I, LumB-I, Basal-I, HER2-I. Two of these were almost entirely similar to the original NMF clusters of Basal-I, and LumA-I. Another cluster highly resembled NMF LumB-I and consistent with Krug et al consisted of 54% luminal A cases (compared to 55% luminal A cases assigned as LumB-I in the original NMF CPTAC clusters by Krug et al). Similar to the original NMF CPTAC clustering composition, the NMF CPTAC HER2-I cluster identified had a mix of Her2-Enriched, luminal A and luminal B breast cancers. Of note, the original Krug et al 2020 study of 122 breast tumors included a majority of luminal A PAM50 subtype (n=57, 47%), followed by basal-like (n=29, 24%), luminal B (n=17, 14%) and Her2-Enriched (n=13, 11%), when compared to the composition of our 08-13 cohort which consisted of a higher number of basal-like (n=73, 42%) and Her2-Enriched (n=62, 36%) cases, but few luminal A cases (n=11, 6%). Despite this, our analysis further demonstrated the existence of subsets enriched for immune response pathways at the proteome level and these included basal-like and Her2-Enriched subtypes. In contrast to our analysis on the 08-13 cohort, these subsets were not captured as separate and defined clusters by the CPTAC analysis. Consistent with our analysis on the 08-13 cohort, stromal pathways were enriched in luminal A tumors and lipid metabolism was enriched within luminal B and Her2-Enriched tumors. (b) **Validation using the Johansson et al 2019 OSLO2 breast cancer landscape cohort:** To validate our findings on the 36 cases of

the 4 main subtypes (9 for each PAM50 type) in the OSLO2 landscape cohort, we performed consensus clustering with the same parameters used in our analysis, using the 775 proteins from the OSLO2 data that overlap with the 1054 mostly highly-variant proteins of our 08-13 cohort. This analysis identified 4 clusters that highly resembled the main consensus core tumor clusters (CoTCs) and their biological functions as reported in Johansson et al. These clusters consisted of CoTC1 (basal-like immune cold), CoTC2 (basal-like immune hot), CoTC3 with few CoTC6 cases (luminal A-enriched) and CoTC6 (luminal B and Her2-Enriched). Importantly, the immune distinctions within the basal-like subtype were entirely reproduced using our highly variant proteins showing that the two basal-like samples of OSL.3EB and OSL.449 (CoTC2) were consistently classified as basal immune hot cluster when compared to other basal cases characterized as basal immune cold. Source data are provided as a Source Data file.

Supplementary Figure 11

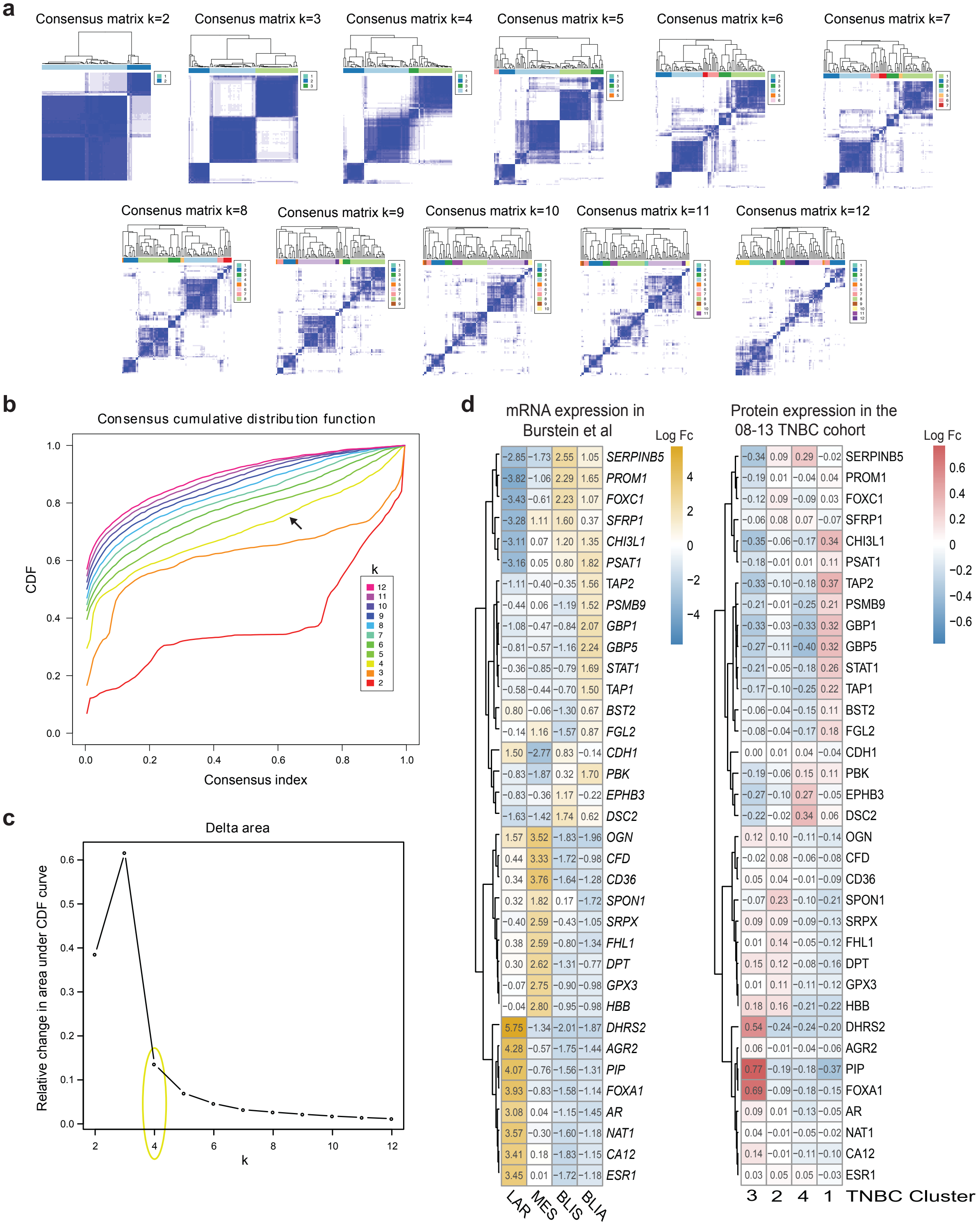

Supplementary Figure 11. Analysis of TNBC cases within the 08-13 cohort.

(a) Consensus matrices exploring the range of 2 to 12 *K*-means clusters for tumors in TNBC cases within the 08-13 cohort using consensus clustering on the 1055 most variant proteins. (b) Consensus CDF area. (c) Delta area showing the relative change in area under the CDF curve. Four robustly segregated groups displayed a clear separation of the clusters based on visual inspection and largest change in area under the CDF curve in delta plot when exploring the range of 2 to 12 *K*-means clusters. (d) Comparative heatmaps showing the log fold change for the expression of individual candidates that characterize each TNBC subgroup by the RNA level in Burstein et al[2] and the protein level in our cohort. Abbreviations: TNBC, triple-negative breast cancer; CDF, cumulative distribution function. Source data are provided as a Source Data file.

Supplementary Figure 12

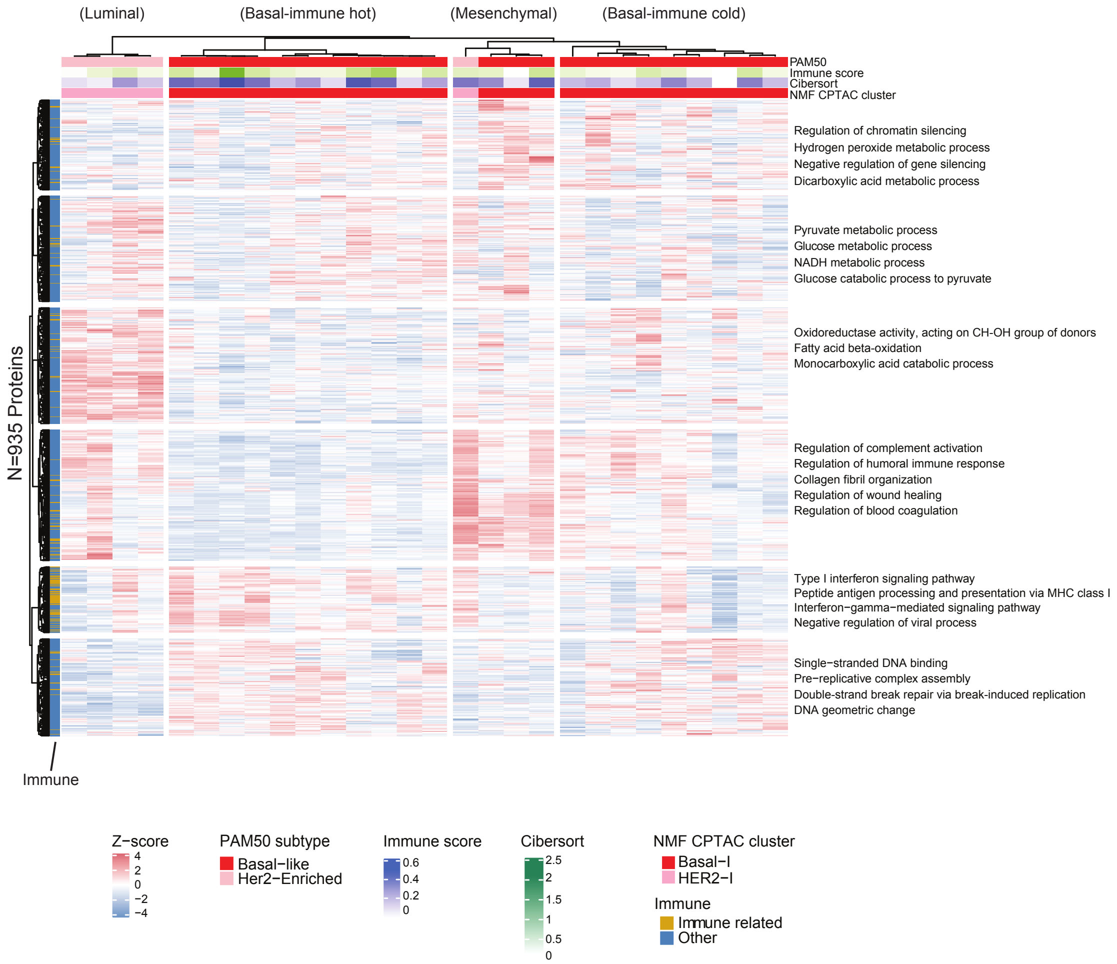

Supplementary Figure 12. Validation of the four proteomic subtypes in TNBC and their biological characteristics using the proteome dataset from Krug et al, 2020. Consensus clustering using the 935 proteins that overlap with the 1055 mostly highly-variant proteins of the 08-13 TNBC subset (n=88) were applied on the proteomic data for a set of 28 TNBC cases included in the breast cancer cohort by Krug et al, 2020. The heatmap illustrates that this analysis reproduced the existence of the four main proteome TNBC subgroups and their biological features of 'luminal-androgen receptor', 'mesenchymal', 'basal-immune suppressed', and 'basal-immune activated'. TNBC, triple-negative breast cancer. Source data are provided as a Source Data file.

Supplementary Figure 13

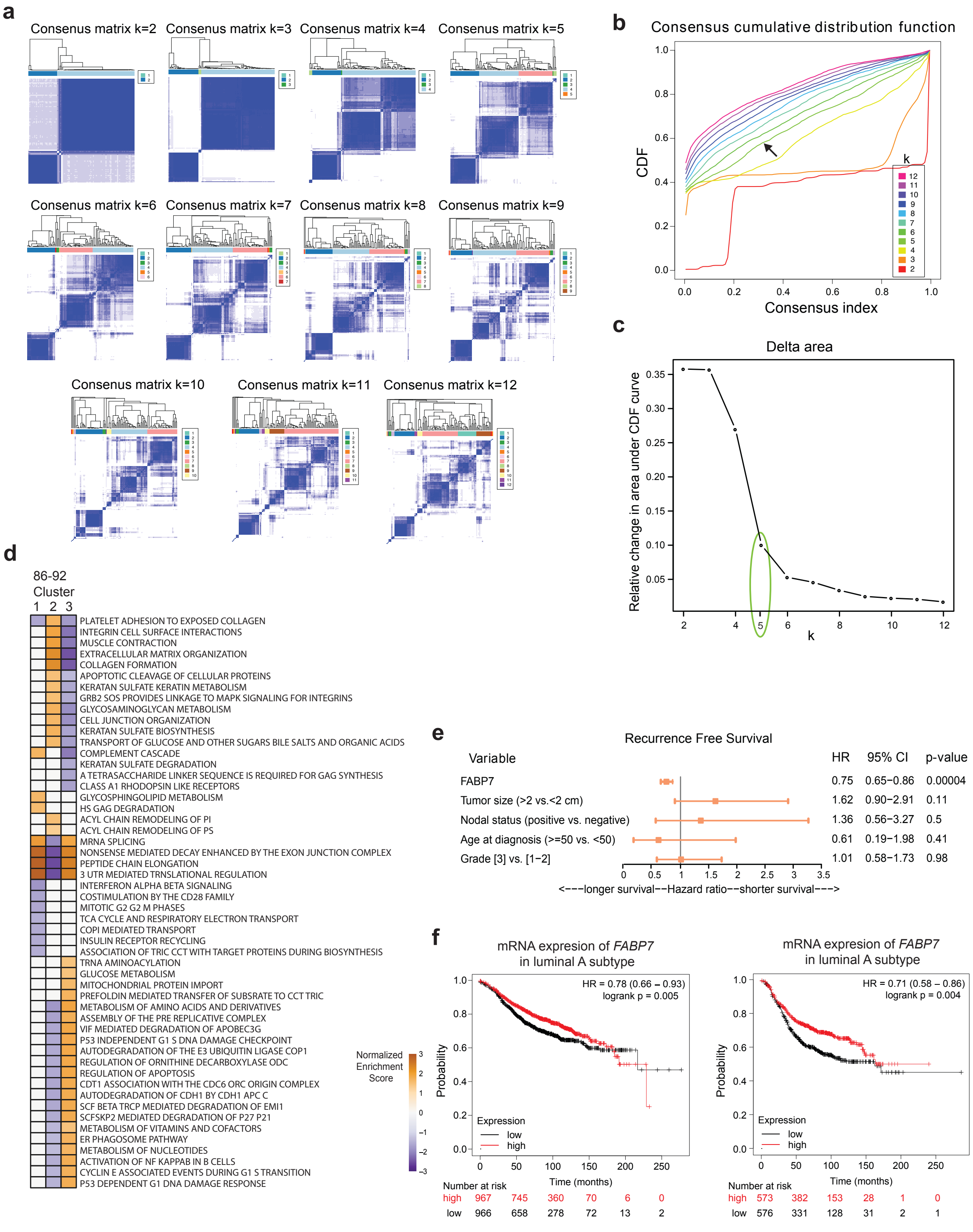

Supplementary Figure 13. Biological and clinical characteristics of cases in the 86-92 cohort.

(a-c) Consensus matrices exploring the range of 2 to 12 *K*-means clusters for tumors in the 86-92 cohort show five robustly segregated groups that display a clear separation of the clusters based on visual inspection of (a) consensus matrices exploring the range of 2 to 12 *K*-means clusters (b) consensus CDF area (c) delta area showing the relative change in area under the CDF curve. Abbreviations: CDF, cumulative distribution function.

(d) Gene set enrichment analysis of selected biological processes with significant differences between the 3 main proteome clusters (adjusted p-value <0.05) in the 86-92 cohort.

(e) Forest plot of the hazard ratio of fatty acid binding protein 7 (FABP7) expression identified as the biomarker most significantly associated with longer RFS on tamoxifen treatment (adjusted p-value = 0.002). The error bars represent 95% confidence interval (CI) as a horizontal line with hazard ratio (HR) result displayed as a plotted box.

(f) Kaplan-Meier survival curves showing the association between mRNA *FABP7* expression and RFS in luminal A and luminal B patients. Plots were generated using the KM-plotter survival tool on breast cancer datasets from Gene Expression Omnibus[3]. Abbreviations: RFS, recurrence free survival. Source data are provided as a Source Data file.

Supplementary Tables

Supplementary Table 2

	**Univariate analysis for RFS**		**Univariate analysis for OS**	
	**HR (95%CI)**	***P***	**HR (95%CI)**	***P***
Cluster 1 vs. others	0.77 (0.36-1.65)	0.49	1.11 (0.54-2.27)	0.78
Cluster 2 vs. others	2.33 (1.29-4.22)	0.005	2.68 (1.45-4.97)	0.001
Cluster 3 vs. others	0.28 (0.11-0.71)	0.008	0.26 (0.09-0.73)	0.01
Cluster 4 vs. others	1.33 (0.70-2.54)	0.39	0.78 (0.36-1.69)	0.53
	**Multivariate analysis for RFS**		**Multivariate analysis for OS**	
	**HR (95%CI)**	***P***	**HR (95%CI)**	***P***
Cluster 1 vs. others	0.79 (0.30-2.08)	0.64	0.81 (0.31-2.12)	0.66
Cluster 2 vs. others	3.02 (1.54-5.91)	0.001	3.07 (1.55-6.10)	0.005
Cluster 3 vs. others	0.28 (0.11-0.73)	0.009	0.23 (0.08-0.65)	0.006
Cluster 4 vs. others	0.94 (0.41-2.12)	0.87	0.97 (0.38-2.46)	0.95
Age at diagnosis<br><50 years vs. ≥50 years	0.78 (0.36-1.67)	0.52	0.41 (0.15-1.10)	0.08
Tumor grade<br>3 vs. [1-2]	0.77 (0.37-1.62)	0.50	1.91 (0.74-4.97)	0.18
Nodal status<br>(Positive vs. negative)	1.74 (0.89-3.38)	0.10	1.67 (0.84-3.36)	0.15
Tumor size<br>(>2 vs. ≤2)	2.08 (1.22-3.54)	0.007	2.13 (1.21-3.75)	0.009
Lympho-vascular invasion<br>(Positive vs. negative)	1.82 (0.94-3.53)	0.07	1.33 (0.65-2.75)	0.44
ER status<br>(Positive vs. negative)	0.42 (0.18-0.99)	0.05	0.78 (0.34-1.79)	0.56
PR status<br>(Positive vs. negative)	1.48 (0.63-3.52)	0.37	1.33 (0.56-3.11)	0.52
Her2 status<br>(Positive vs. negative)	1.37 (0.74-2.54)	0.32	1.10 (0.56-2.15)	0.80

Supplementary Table 2: Univariate and multivariate analysis for the different proteome clusters and clinicopathological characteristics in the 08-13 cohort. Results are derived from Cox regression models and stratified log-rank tests with the endpoints of RFS and OS for a multivariate analysis adjusted for clinicopathological variables of pathological tumor size, nodal status, grade, age at diagnosis, lymphovascular invasion, hormone and Her2 receptor status. Abbreviations: RFS, recurrence free survival; OS, overall survival.

Supplementary Table 3

**Characteristic**	**IHC Validation cohort (n=176)**
**Age at diagnosis (median)**	53 years
**Tumor size (median)**	2 cm
**Tumor grade**	
1, 2	44 (25%)
3	127 (72%)
Missing	5 (3%)
**Nodal status**	
Negative	105 (60%)
Positive	66 (37%)
Missing	5 (3%)
**IHC subtype**	
Luminal ([ER+ or PR+])	69 (39%)
ER-, PR-, HER2+	32 (18%)
ER-, PR-, HER2-	71 (40%)
Missing	4 (8%)
**Disease specific death**	
No	134 (76%)
Yes	35 (20%)
Missing	7 (4%)
**CD8 iTILs**	
<1%	42 (24%)
≥1%	129 (73%)
Missing	5 (3%)
**TAP1/HLA-DQA1 IHC groups**	
TAP1 high /HLA-DQA1 high	35 (20%)
TAP1 low /HLA-DQA1 high	22 (13%)
TAP1 high /HLA-DQA1 low	50 (28%)
TAP1 low /HLA-DQA1 low	65 (37%)
Missing	4 (2%)

Supplementary Table 3: Characteristics of the independent IHC validation cohort.

Supplementary Table 5

	Univariate analysis for RFS		Univariate analysis for OS	
	**HR (95%CI)**	***P***	**HR (95%CI)**	***P***
86-92 Cluster 1 vs. others	1.47 (0.85-2.53)	0.17	1.08 (0.68-1.73)	0.74
86-92 Cluster 2 vs. others	0.82 (0.44-1.54)	0.54	0.79 (0.46-1.34)	0.38
86-92 Cluster 3 vs. others	0.79 (0.43-1.42)	0.41	1.14 (0.70-1.84)	0.60
	**Multivariate analysis for RFS**		**Multivariate analysis for OS**	
	**HR (95%CI)**	***P***	**HR (95%CI)**	***P***
86-92 Cluster 1 vs. others	1.37 (0.74-2.55)	0.32	1.10 (0.60-2.00)	0.76
86-92 Cluster 2 vs. others	0.86 (0.44-1.67)	0.65	0.87 (0.44-1.72)	0.69
86-92 Cluster 3 vs. others	0.83 (0.43-1.61)	0.59	1.03 (0.53-2.01)	0.93
Tumor grade<br>3 vs. [1-2]	1.09 (0.60-1.98)	0.78	1.24 (0.73-2.11)	0.44
Nodal status<br>(Positive vs. negative)	1.28 (0.52-3.16)	0.59	1.07 (0.55-2.08)	0.85
Tumor size<br>(>2 vs. ≤2)	1.63 (0.91-2.93)	0.10	2.09 (1.27-3.42)	0.003
Lympho-vascular invasion<br>(Positive vs. negative)	1.84 (0.87-3.88)	0.11	1.40 (0.79-2.48)	0.25

Supplementary Table 5: Univariate and multivariate analysis for the three main proteome clusters and clinicopathological characteristics in the 86-92 cohort. Results are derived from Cox regression models and stratified log-rank tests with the endpoints of RFS and OS for a multivariate analysis adjusted for clinicopathological variables of pathological tumor size, nodal status, grade, age at diagnosis and lymphovascular invasion. Abbreviations: RFS, recurrence free survival; OS, overall survival.

Supplementary References

1 Cheung, T. K. *et al.* Defining the carrier proteome limit for single-cell proteomics. *Nat Methods* **18**, 76-83, doi:10.1038/s41592-020-01002-5 (2021).

2 Burstein, M. D. *et al.* Comprehensive genomic analysis identifies novel subtypes and targets of triple-negative breast cancer. *Clin Cancer Res* **21**, 1688-1698, doi:10.1158/1078-0432.CCR-14-0432 (2015).

3 Györffy, B. *et al.* An online survival analysis tool to rapidly assess the effect of 22,277 genes on breast cancer prognosis using microarray data of 1,809 patients. *Breast Cancer Res Treat* **123**, 725-731, doi:10.1007/s10549-009-0674-9 (2010).
