# Supplementary material for: Proteomic analysis of archival breast cancer clinical specimens identifies biological subtypes with distinct survival outcomes
Source: Nat Commun. 2022 Feb 16;13:896. doi: 10.1038/s41467-022-28524-0 (PMC8850446; doi:10.1038/s41467-022-28524-0)
Supplement: Supplementary file 3 — Description of Additional Supplementary Information [file 41467_2022_28524_MOESM3_ESM.pdf]

Description of Additional Supplementary Information

Supplementary Data 1: Breast Cancer Proteomics Meta Data. Supplementary Data 1a includes the study design, Supplementary Data 1b includes the isoDoping peptide library, Supplementary Data 1c includes the clinical and pathological characteristics for cases included in the correlative analyses of the study cohorts. Related to Figure 1 and Supplementary Figures 1-2.

Supplementary Data 2: Biological differences between the proteome clusters of the 08-13 cohort. Supplementary Data 2a includes the expression of highly variant proteins across samples. Supplementary Data 2b includes the association between proteome clusters and PAM50 subtype. Supplementary Data 2c includes results of the differential protein expression analysis between each cluster compared to other clusters. Supplementary Data 2d-2g includes the pathway enrichment analysis between each proteome cluster compared to other clusters. Supplementary Data 2h includes the distribution of clinico-pathological characteristics across the different proteome clusters in the 08-13 cohort. Related to Figures 2-3 and Supplementary Figures 5-6.

Supplementary Data 3: Biological and clinical data analysis of the TNBC cohort in the 08-13 cohort. Supplementary Data 3a includes the association between TNBC clusters and PAM50 subtype. Supplementary Data 3b includes the results of differential protein expression analysis between each TNBC cluster compared to other clusters. Supplementary Data 3c-3f includes the pathway enrichment analysis for each TNBC cluster compared to other TNBC clusters. Supplementary Data 3g includes the association between TNBC clusters and the 08-13 proteome

clusters. Supplementary Data 3h-3i includes the survival analysis for individual proteins and their characteristics according to TNBC cluster. Related to Figures 5-6 and Supplementary Figure 11.

Supplementary Data 4: Biological differences between the main three proteome clusters of the 86-92 cohort. Supplementary Data 4a includes the association between the 86-92 clusters and PAM50 subtype. Supplementary Data 4b includes the results of differential protein expression analysis between each 86-92 cluster compared to other clusters. Supplementary Data 4c-4e includes the pathway enrichment analysis for each 86-92 cluster compared to other clusters. Supplementary Data 4f includes the recurrence free survival analysis for individual proteins. Supplementary Data 4g includes the distribution of clinico-pathological characteristics across the three main proteome clusters in the 86-92 cohort. Related to Figure 7 and Supplementary Figure 13.
