# Supplementary material for: Proteomic analysis of archival breast cancer clinical specimens identifies biological subtypes with distinct survival outcomes
Source: Nat Commun. 2022 Feb 16;13:896. doi: 10.1038/s41467-022-28524-0 (PMC8850446; doi:10.1038/s41467-022-28524-0)
Supplement: Supplementary file 8 — Reporting Summary [file 41467_2022_28524_MOESM8_ESM.pdf]

nature research

Corresponding author(s): Gregg B. Morin

Last updated by author(s): Dec 11, 2021

Reporting Summary

Nature Research wishes to improve the reproducibility of the work that we publish. This form provides structure for consistency and transparency in reporting. For further information on Nature Research policies, see our Editorial Policies and the Editorial Policy Checklist.

Statistics

For all statistical analyses, confirm that the following items are present in the figure legend, table legend, main text, or Methods section.

n/a	Confirmed	
☐	☒	The exact sample size (*n*) for each experimental group/condition, given as a discrete number and unit of measurement
☐	☒	A statement on whether measurements were taken from distinct samples or whether the same sample was measured repeatedly
☐	☒	The statistical test(s) used AND whether they are one- or two-sided<br>*Only common tests should be described solely by name; describe more complex techniques in the Methods section.*
☐	☒	A description of all covariates tested
☐	☒	A description of any assumptions or corrections, such as tests of normality and adjustment for multiple comparisons
☐	☒	A full description of the statistical parameters including central tendency (e.g. means) or other basic estimates (e.g. regression coefficient) AND variation (e.g. standard deviation) or associated estimates of uncertainty (e.g. confidence intervals)
☐	☒	For null hypothesis testing, the test statistic (e.g. *F*, *t*, *r*) with confidence intervals, effect sizes, degrees of freedom and *P* value noted<br>*Give P values as exact values whenever suitable.*
☒	☐	For Bayesian analysis, information on the choice of priors and Markov chain Monte Carlo settings
☐	☒	For hierarchical and complex designs, identification of the appropriate level for tests and full reporting of outcomes
☐	☒	Estimates of effect sizes (e.g. Cohen's *d*, Pearson's *r*), indicating how they were calculated

Our web collection on statistics for biologists contains articles on many of the points above.

Software and code

Policy information about availability of computer code

Data collection	Mass spectrometry data were acquired on an Orbitrap Fusion using Xcalibur software (ver 4.3)
Data analysis	Proteomics data were searched with Proteome Discoverer (ver 2.4).<br>Downstream analysis was performed in R statistical software (version 3.5.0).<br>Several R packages were used for data wrangling, analysis and visualization as follows:<br>ConsensusClusterPlus_1.46.0, umap_0.2.3.1, plyr_1.8.4, cleaver_1.20.0, gprofiler2_0.1.9, GGally_1.4.0, tidyr_1.1.3, dplyr_1.0.7, ggalluvial_0.9.1, ggrastr_0.2.1, survminer_0.4.3, ggpubr_0.1.8, RegParallel_1.0.0, survival_3.2-3,ComplexHeatmap_1.99.6, PECA_1.18, ggrepel_0.8.0, ggplot2_3.3.5, pheatmap_1.0.1, stringr_1.4.0, data.table_1.14.0, fgsea_1.8.0, writexl_1.0, tidyverse_1.3.0<br><br>R code used for proteomics data processing and analysis is available at GitHub through the following link https://github.com/glnegri/brca. The following Zenodo link can be used for citation: https://doi.org/10.5281/zenodo.5873584. Citation: Asleh K, Negri G, Spencer Miko SE, et al. Proteomic analysis of archival breast cancer clinical specimens identifies biological subtypes with distinct survival outcomes. Github, doi:10.5281/zenodo.5873584 (2022).

For manuscripts utilizing custom algorithms or software that are central to the research but not yet described in published literature, software must be made available to editors and reviewers. We strongly encourage code deposition in a community repository (e.g. GitHub). See the Nature Research guidelines for submitting code & software for further information.

Data

Policy information about availability of data

All manuscripts must include a data availability statement. This statement should provide the following information, where applicable:

- Accession codes, unique identifiers, or web links for publicly available datasets
- A list of figures that have associated raw data
- A description of any restrictions on data availability

The mass spectrometry proteomics data have been deposited to the ProteomeXchange Consortium via the PRIDE partner repository with the dataset identifier/accession code PXD024322 (“PXD024322 [https://www.ebi.ac.uk/pride/]”).
Mass spectrometry data were searched against the UniProt Human proteome (03/08/2018 release, 20387 sequences) database. Gene set enrichment analysis was performed using annotated signatures: GO term signature (‘c5.all.v6.0’) as described in Subramanian et al (available online) – unique identifier: https://www.gsea-msigdb.org/gsea/msigdb/index.jsp and
REACTOME pathways (‘c2.cp.reactome.v6.0’) as described in Subramanian et al (available online) – unique identifier: https://www.gsea-msigdb.org/gsea/msigdb/index.jsp.
An anonymized data file containing characteristics of the study datasets, proteome clusters, and protein scores, used and analyzed in this study can be found in Supplementary Data 1 and the Source Data file of this manuscript.
Images from immunohistochemistry slides of tissue microarrays used in the study coded as 11-012 and 14-004 are available online for public access via the website of Genetic Pathology Evaluation Center – unique identifier: http://www.gpec.ubc.ca/prot. Clinical data for the patients included in this study are not publicly available per policy to protect patient privacy. Clinical data access including de-identified individual patient characteristics and survival outcomes can be made available for qualified researchers on a request that does not include revelation of identifiable patient information through the Genetic Pathology Evaluation Centre and Breast Cancer Outcome Unit of BC Cancer, upon completion of a Data Transfer Agreement and confirmation of ethical approval. This clinical information would include the patient characteristic variables as presented in Supplementary Data 2h and 4g. Requests or queries should be directed to the corresponding author. Queries for data access will be answered within a time frame required to ensure high quality assessment and coordination of the proposed collaborative work and a first response can be provided within approximately two weeks.
This study involved the collection and analysis of data from multiple publicly-available datasets. The CPTAC publicly-available breast cancer dataset used in this study are available in the Supplementary Information of Krug et al (available online) – unique identifier: https://doi.org/10.1016/j.cell.2020.10.036. The OSLO2 publicly-available breast cancer dataset used in this study are available in the Supplementary Data of Johansson et al (available online) – unique identifier: https://www.nature.com/articles/s41467-019-09018-y#Sec15. Survival analysis for FABP7 mRNA expression was performed using the previously-established KMplotter analysis platform (available online) – unique identifiers: (https://kmplot.com/analysis/) and (10.1007/s10549-009-0674-9). The remaining data are available within the Article, Supplementary Data or Source Data files or as deposited at PRIDE.
The source data including detailed information on proteins and peptides used in the quantification, analysis and data representation are provided as a source data file in this article.

Field-specific reporting

Please select the one below that is the best fit for your research. If you are not sure, read the appropriate sections before making your selection.

☒ Life sciences ☐ Behavioural & social sciences ☐ Ecological, evolutionary & environmental sciences

For a reference copy of the document with all sections, see nature.com/documents/nr-reporting-summary-flat.pdf

Life sciences study design

All studies must disclose on these points even when the disclosure is negative.

Sample size

Sample size of 300 tumor samples included in the study were determined based on the inclusion of 75 representative samples from each PAM50 tumor subtype. This number is enough powered to allow the identification of biological clusters and discrimination of clinical outcomes. 38 normal samples were selected to allow the inclusion of one normal sample in each batch run that were 38 in total.

Data exclusions

In order to remove samples with low signal, we set a minimum filter (2e06) on total Signal/Noise ratio and removed 14 samples that did not pass this quality control threshold from the analysis.

Replication

Validation of results observed with immunohistochemical biomarkers and clinical outcomes was performed on an independent set of 176 breast cancer cases with similar clinicopathological characteristics to the 08-13 cohort to reproduce our findings.
Validation of results observed with the proteomics analysis for the 08-13 cohort and the triple negative breast cancer subset was performed using two available published proteomics breast cancer datasets of (a) CPTAC breast tumor cohort by Krug et al (b) OSLO2 breast cancer landscape cohort by Johansson et al to reproduce our findings.

Randomization

A random selection of two samples from each main PAM50 subtype and 1 sample from normal reduction mammoplasty was followed in the design of each 11-plex batch in this study that were run to include a normal reduction mammoplasty sample (TMT11 126), 2 samples of each of the tumor PAM50 subtypes (luminal A (TMT11-127N, TMT11-127C), luminal B (TMT11-128N, TMT11-128C), basal-like (TMT11-129N, TMT11-129C), Her2-Enriched (TMT11-130N, TMT11-130C)), a “SuperMix” control consisting of 13 cancer cell lines (TMT11-131N), and a pooled internal standard (PIS) made up of aliquots from the tumor and normal samples plus isoDoping peptides (TMT11-131C).
In addition, data collection for all the final 38 samples for each single fraction obtained in mass spectrometry was done in a randomized order to reduce batch effects over the course of data acquisition.

Blinding	All immunohistochemical biomarkers were independently scored by pathologists blinded to the proteomics data and clinical outcome data included in this study.

Reporting for specific materials, systems and methods

We require information from authors about some types of materials, experimental systems and methods used in many studies. Here, indicate whether each material, system or method listed is relevant to your study. If you are not sure if a list item applies to your research, read the appropriate section before selecting a response.

Materials & experimental systems

n/a	Involved in the study
☐	☒ Antibodies
☒	☐ Eukaryotic cell lines
☒	☐ Palaeontology and archaeology
☒	☐ Animals and other organisms
☐	☒ Human research participants
☒	☐ Clinical data
☒	☐ Dual use research of concern

Methods

n/a	Involved in the study
☒	☐ ChIP-seq
☒	☐ Flow cytometry
☒	☐ MRI-based neuroimaging

Antibodies

Antibodies used	HLA-DQA1 IHC antibody Abcam Cat# ab128959<br>IFIT2 IHC antibody Abcam Cat# ab113112<br>S100A8 IHC antibody R&D Systems Cat# MAB4570<br>TAP1 IHC antibody Proteintech Cat# 11114-1-AP<br>CD8 IHC antibody Dako Cat# M7103
Validation	Serial 4μm sections from each tissue microarray were stained for S100A8, TAP1, IFIT2, HLA-DQA1 and CD8 according to the Discovery XT semi-automated immunostainer protocol (Ventana medical Systems Inc. Tucson, AZ USA). The following antibodies were used: anti-S100A8 monoclonal mouse primary antibody (clone 749916, dilution 1:1000, R&D Systems, cat# MAB4570); anti-TAP1 polyclonal rabbit primary antibody (dilution 1:250, Proteintech, cat# 11114-1-AP); anti-IFIT2 polyclonal rabbit primary antibody (dilution 1:1000, Abcam, cat# ab113112); anti-HLA-DQA1 monoclonal rabbit primary antibody (clone [EPR7300], dilution 1:500, Abcam, cat# ab128959); anti-CD8 monoclonal mouse primary antibody (clone [C8/144B], dilution 1:50, Dako, cat# M7103).<br>Slides underwent antigen retrieval with standard Cell Conditioning 1 (Ventana Medical Systems) followed by 60 min of primary antibody incubation with heat, and detected using a DAB Map Detection Kit (Ventana Medical Systems). Slides were then incubated with a secondary antibody (Ventana universal secondary antibody) for an additional 32 min.

Human research participants

Policy information about studies involving human research participants

Population characteristics	Tumor cohorts: The current study included 300 archival FFPE tissues corresponding to primary tumor excision specimens of patients diagnosed with invasive breast cancer in the periods of "2008-2013" (n=178; 08-13 cohort) and "1986-1992" (n=122; 86-92 cohort). These specimens were assembled from different centers across the province of British Columbia and retained at Vancouver General Hospital, Canada.<br>Normal cohort: A set of normal samples were sourced from 38 healthy women who were referred to plastic surgery at Vancouver General Hospital and UBC hospital for reduction mammoplasties in the period January 2015 to September 2017. The median age of women at time of surgery was 42 years old and ranged between 19-61 years old.<br>IHC validation cohort: A tissue microarray for an independent set of 176 breast cancer cases was used to validate observations on the 08-13 cohort for the key protein immunohistochemical biomarkers. This validation cohort was sourced from breast cancer women who were referred to the BC Cancer between the years 2005-2009 and had similar clinicopathological characteristics to the 08-13 cohort. The median follow-up for the IHC validation cohort was 10 years and patients were treated in accordance with contemporary guidelines.
Recruitment	No patients were recruited as part of this study.
Ethics oversight	This study was approved in accordance with the ethical standards of the institutional board of the University of British Columbia and BC Cancer (approval number: H17-01207). All patients had signed a written informed consent to allow the use of their tumor tissue for future study–related research purposes. The approval for the subsequent use of these previously assembled patient specimens for this proteomics study was obtained under a waiver of informed consent policy, per the Canadian Tri-Council Policy Statement: Ethical Conduct for Research Involving Humans-TCPS 2. Only de-identified, coded study ID numbers were provided, and no participant compensation is associated with this study.

Note that full information on the approval of the study protocol must also be provided in the manuscript.
